# Plastic neuroscience: studying what the brain cares about

**DOI:** 10.3389/fnhum.2014.00176

**Published:** 2014-04-23

**Authors:** Joseph Dumit

**Affiliations:** Science and Technology Studies, University of California, DavisDavis, CA, USA

**Keywords:** critical neuroscience, history of neuroscience, experimental design, cognitive neuroscience, plasticity, play

## Abstract

Drawing on Allan Newell's “You can't play 20 questions with nature and win,” this article proposes that neuroscience needs to go beyond binary hypothesis testing and design experiments that follow what neurons care about. Examples from Lettvin et. al. are used to demonstrate that one can experimentally play with neurons and generate surprising results. In this manner, brains are not confused with persons, rather, persons are understood to do things with their brains.

Allan Newell's infamous 1973 commentary challenged the design of cognitive neuroscience experiments: “You can't play 20 questions with nature and win.” Delivered at a conference attended by many titans of psychology—e.g., Mike Posner, Herbert Simon, Lynn Cooper, Roger Shepard—Newell began,

I am a man who is half and half. Half of me is half-distressed and half-confused. Half of me is quite content and clear on where we are going. We have just listened to a sample of the best work in current experimental psychology… and almost all of the material shown here serves to further a view of man as a processor of information, agreeing with my current theoretical disposition. Half of me is ecstatic. …

Still, I am distressed (because imagining 40 more years of these papers on new aspects of the problems), where will psychology then be? Will we have achieved a science of man adequate in power and commensurate with his complexity? And if so, how will this have happened via these papers… ? Or will we be asking for yet another quota of papers in the next dollop of time? (Newell, 1973, 1).

For Newell, you can't play 20 questions with nature and win because asking binary yes/no questions generates many papers but settles almost nothing. It is inventive of methods but stops too early. One “frame(s) a general question, hopefully binary, that can be attacked experimentally. Having settled that bits-worth, one can proceed to the next. The policy appears optimal—one never risks much, there is feedback from nature at every step, and progress is inevitable. Unfortunately, the questions never seem to be really answered, the strategy does not seem to work^1^.” Newell effectively describes an entrenched “thought style^2^,” that forecloses real answers. Correlations between brain measurements and cognitive psychology performances not only don't add up to causation, they have a good chance of diverting scientists from it, precisely by seeming like a stopping point. The main risk for neuroscientists is not that they become wrong, but that they become trivial:

Every time we find a new phenomenon… we produce a flurry of experiments to investigate it… and the combinational variations flow from our experimental laboratories. (Yet by only varying issues and binaries,) matters simply become muddier and muddier as we go down through time. Thus, far from providing the rungs of a ladder by which psychology gradually climbs to clarity, this form of conceptual structure leads rather to an ever increasing pile of issues, which we weary of or become diverted from, but never really settle. (Newell, 1973, 8)^3^.

In order to diagnose the core problem facing cognitive studies, Newell looked at the crucial but ambiguous use of flow diagrams in designing and confirming hypotheses. While they appeared to be analogies of computers, they were in fact sloppy and incomplete, because a computer is only useful while running, and in order to run, it needs a language, with operations and syntax. Each running computer has an operating system, what Newell called a “control structure,” that is essential to its being a running computer.

Much of the new progress in the experimental analysis of the information processing of humans has eschewed attention to the control structure. The best example… is the deservedly well-known paper by Atkinson and Shiffrin (1968) entitled: “Human Memory: A proposed system and its control processes.” The model of memory is there all right, and is applied to a number of tasks with quantitative precision. However, the control structure is completely absent and is used as a deus ex machina to concoct separate models for each task. Criticism is not directed at that justly influential piece of work. But it does illustrate well the current state of the theoretical art. As long as the control structure—the glue—is missing, so long will it be possible to suggest an indefinite sequence of alternative possibilities for how a given task was performed, hence to keep theoretical issues from becoming settled. (Newell, 15).

For Newell, the ambiguous nature of the flow charts was the heart of the neuroscience thought style and a key factor in its productivity (of articles) and its weakness (in really learning something). Newell's concern was that the notion of circuits and modules is not inherently wrong about brains or minds, but is based on the wrong idea of computers! As a programmer of early computers, he starts from the same premise as Norbert Wiener, “The brain… is not the complete analog of the computing machine but rather the analogue of a single run on such a machine” (Weiner, 1948, 121)^4^. The brain must be modeled not just in hardware or even software, but running software, the kind that does not always boot up the same way.

“Explanations must come to an end somewhere.” Thus philosopher Ludwig Wittgenstein called attention to the historical cultural practices by which people within a thought style behave with regard to knowledge (Wittgenstein, 2010)^5^. Some answers cause us to ask more questions, while others somehow satisfy us, in the sense that we no longer ask, “But why is that?” Why are we satisfied when neuroscience finds a correlation between a behavior and brain activation? Why isn't that satisfaction most perplexing? In my book, *Picturing Personhood: Brain Scans and Biomedical Identity* (Dumit, 2004), I queried: if a strong correlation was found between a diagnosis of schizophrenia and blood flow to the big toe, we would immediately ask, why? But even if a less strong correlation was found between the diagnosis and bloodflow in a small region of the brain, one would publish the results and refer to a “region *mediating* schizophrenia” as having finally been found.

Perhaps we can imagine a method that would instead take correlation as the starting point for hypotheses. In a recent neuroeconomics paper, “Neural Signatures of Economic Preferences for Risk and Ambiguity,” Huettel et al. (2006) argue:

Within pIFS (posterior inferior frontal sulcus), a dramatic effect of ambiguity was observed compared with decisions involving risk. At the outset of each trial, when subjects considered their options and made a choice, activation was several times greater for decisions involving ambiguity than decisions involving risk. … We conclude that activation in pIFS, unlike in other regions, mediates processes that counter cognitive impulsiveness in decision making. (767 and 770).

While the paper uses this correlation to argue for *mediation*, perhaps we should not treat this like a conclusion, but instead as the beginning of a hypothesis rhizome. While there is a correlation, the signal is quite noisy and variable across persons. Should we not then ask, what behavior could be induced in participants to really and reliably light up this region (pIFS)? In other words, let's assume that the weak but statistically significant correlation is not in the brain region, but that the brain region is suggestively yet poorly defined by this notion of “ambiguity and cognitive impulsiveness.” Perhaps the brain region is doing something quite different and we contemporary humans are improvisationally appropriating it for the purposes of distinguishing ambiguity. Can we use the region's lighting up to figure out a better description of the *brain's* behavior?

A precursor to this type of questioning can be found in that famous neuroscience study: “What the Frog's Eye tells the Frog's Brain.” Lettvin et al. (1959) were trying to figure out what particular neurons cared about, “we analyze the activity of single fibers in the optic nerve of a frog. Our method is to find what sort of stimulus causes the largest activity in one nerve fiber and then what is the exciting aspect of that stimulus such that variations in everything else cause little change in the response.” Through lots of creative trials, they found neurons that responded to small things that moved like flies and ignored stationary things and ensembles of things that moved together. But they didn't stop. In a follow-up paper, “Two Remarks on the Visual System of the Frog,” (Lettvin et al., 1960) they offered:

Perhaps we had better say a word about our experimental procedure,.. Our stimuli consisted of silhouettes of different size and shape… moved against the background by means of magnets… Response of a nerve fiber was measured roughly by frequency and duration of firing. *We took no records except those needed to illustrate our papers. Our question was not how great the response was to one or another manipulation, but rather which visual events produced greatest response and which produced least, and what aspect of the image could be varied without changing the response*. We dealt with our own listening to the patterns of nerve spikes as the measure of extremes, just as one does with an a–c bridge. (Emphasis added).

For contemporary laboratories, this is not really a method. They are taking up a playful relationship to the nerve fibers themselves, asking, What can we do to the frog that will maximize the signal produced by particular nerves? Rather than staying with a method, they were exploring whether they could let the nerve fibers induce in the researchers a nerve-specific behavior—anti-methodology by design:

We took no records except those needed to illustrate our papers… Early in our work we found that taking records hindered rather than helped this kind of research by leading to premature standardization of method.

They are calling for *a plastic neuroscience*, one whose methods remain adaptable to what is called forth by the brain. This is an abductive method, neither deductive nor inductive, but one in which the method is invented retroactively as if abducted or possessed by the object one is trying to study^6^.

When I have presented this abductive approach to senior researchers as well as to undergraduate neuroscience majors, I received the same response, “Well that might be more ecologically valid… but we couldn't run a lab that way” (See Gibson, 1979). But consider Lettvin et al.'s startling discovery:

“Sameness” Neurons

It is a bit embarrassing to present the following description so batrachomorphically, but at least it reflects what we have found so far. Every such cell, in fact, acts so complexly that we can hardly describe its response save in terms ordinarily reserved for animal behavior… It is silent. We bring in a (small) dark object… and at a certain point in its travel, almost anywhere in the field, the cell suddenly “notices” it. Thereafter, wherever that object is moved it is tracked by the cell. Every time it moves, with even the faintest jerk, there is burst of impulses that dies down to a mutter that continues as long as the object is visible. If the object is kept moving, the bursts signal discontinuities in the movement, such as the turning of corners, reversals, and so forth, and these bursts occur against a continuous background mutter that tells us the object is visible to the cell. When the target is removed, the discharge dies down. If the target is kept stationary for about 2 min, the mutter also disappears. Then one can sneak the target around a bit, slowly, and produce no response, until the cell “notices” it again and locks on. Thereafter, no small or slow movement remains unsignaled.

..There is also (we put this matter very hesitantly) an odd discrimination in these cells, which, though we would not be surprised to find it in the whole animal, is somewhat startling in single units so early behind the retina… Suppose we have two similar targets… It seems to attend one or the other, A or B; its output is not a simple combination of the responses to both.

Here is a neuronal practice of attention—how a neuron (not a frog, or a person) attends to something. This is not necessarily what a human might want from attention (a gamer or hunter or student might want something else, and they may be able to recruit such a neuron into desired forms of attention). Through their abductive playing with the neurons, the researchers thoroughly surprised themselves. Neurons as animals. Then they tried to verify their finding:

These descriptions are provisional and may be too naturalistic in character. However, we have examined well over a hundred cells and suspect that what they do will not seem any simpler or less startling with further study… Of course if one were to perform the standard gestures, such as flashing a light at the eye, probably the cells could be classified and described more easily. However, it seems a shame for such sophisticated units to be handled that way—roughly the equivalent of classifying people's intelligence by the startle response.

Soberly, they realize that had they standardized their method early on, they would have gotten a perfectly acceptable (and publishable) result but it would have been one that conformed the neurons to the method, rather than the other way around.

So how can we push this further and prevent having an experimental design that is satisfied too easily: get a significant correlation and publish. Can we imagine abductively *playing* with the pIFS or oxytocin, using fMRI and TCS to figure out what they are really up to? And in a way that might cause real surprise, even call into question the current configuration of concepts of “preferences,” “risk,” and “ambiguity”? Historians and anthropologists of science have shown that in different times and cultures there are stunningly different notions of rationality, risk, subjectivity, sexuality, personhood, and dangerousness^7^. Given this variability, why should a brain structure necessarily do what a contemporary person does?

To expand on the possibility of learning through abductive play, consider the famous Halle Berry neuron: a fascinating experiment in which a single neuron fired on pictures of Halle Berry, caricatures of her, catwoman, and the letters “HALLE BERRY” (Quiroga et al., 2005). But why did the experimenters stop with well-known examples of that category?^8^ Wouldn't the main question be to figure out where the edges of the category were *for that neuron?* Were there really no surprises in what the neuron reacted to or didn't react to? Couldn't this have been a way to figure out what a neuron thinks a category, such as “Halle Berry,” is?

What a neuron thinks a category is—this is the radical promise of neuroscience that I think we could pursue: we can ask a person what a category is, but then we can also test a brain region, a module, a circuit, skin (through GSR measures), and individual neurons for what of these biological units “thinks” a category is. In each case, we might get different and even surprising answers^9^.

We can then ask: given that a neuron's category is configured in this way, how does a person today use that type of neuron to have the sort of categories that our culture today makes use of? Ramachandran (1998, 2003) shows that even after we figure out what a bit of brain might be doing, there still remains the question of what the human does with that bit. Whether a signal is linked to an amputated limb, or indicates a person isn't familiar (as in Capgras syndrome), Ramachandran looks at how humans improvise a coherent world such that there is a phantom limb, or an alien substituted for one's father. I emphasize “improvise” to note that the coherence that results does not follow directly from brain but is added by person or culture.

Ramachandran proposes that much of human life, culture, is about exaptation, “a mechanism that originally evolved for one function and then provided the opportunity for something very different… to evolve.” “Something very different” means that knowing the origin of behavior or belief X is interesting, but it does little if anything to explain how it is being used now.

Brains and neurons need to be approached this way, as potentially alien things that each historical period and culture has exapted to make its own coherence. From this perspective, stopping at a correlation between a currently coherent behavior and a neural module is ethnocentric!

To sum up then,

1. Why should a brain region do anything like what a person does?
2. Correlations are not stopping points but starting points.
3. Play with method to maximize the region to understand what *it* does.
4. Then investigate how a person improvises activity *with* that brain structure.

In this manner we can render neuroscience plastic and learn what our brains care about.

## Conflict of interest statement

The author declares that the research was conducted in the absence of any commercial or financial relationships that could be construed as a potential conflict of interest.

## Footnotes

^1^Negative and positive appreciations of Newell's claims may be found in Kosslyn (2006) and Gigerenzer and Regier (1996).

^2^“Thought styles” were defined by historian of medicine Ludwig Fleck, who found that different disciplines had distinct ways of assessing publishable results (Fleck, 1981).

^3^Much more should be investigated concerning the synergy between this thought style and the economics of academic audit and publish or perish (Strathern, 2000; Biagioli and Galison, 2003; Mirowski, 2011).

^4^Thinking through the implications of this for contemporary cognitive neuroscience, we might go back to one of its launching points, Plans and the Structure of Behavior, by Miller et al. (1960), which is a sustained meditation on what we might learn about the mind by contemplating its uneasy relation to the model of brain-as-computer. While there is not enough space to investigate that book here, it would be a good starting point for asking the question of how neuroscience might put economics into variation.

^5^Wittgenstein called these thought styles, “forms of life.”

^6^Abduction as method was coined by Peirce (1903) in his fifth and sixth Lectures on Pragmatism (Peirce, 1931–1958, 5.145). See Doyle (2003). Hustak and Myers (2012) describe Darwin's methodological possession by orchids. For contemporary Artificial Intelligence researchers, abduction is a process of inferring or evolving a hypothesis from observations.

^7^For instance, historians Foucault (1978); Castel (1991); Laquer (1990), and anthropologists such as Verran (2001) and Strathern (2004).

^8^There are of course very practical problems with the production of experiments, including the length of time a subject can have their neurons measured, finding the neurons in the first place, etc. And many experimenters have much more ambitious goals in mind. My critique here asks after the reason why the step that gets rewarded by grants and published is the binary hypothesis testing one.

^9^Or scaling up, we might ask what a family, a group, a culture, a nation thinks “trust” or “risk” or a category are.
